# Microstructure and Mechanical Properties of Toughened Seven-Wire Electrogas Welding

**DOI:** 10.3390/ma18071581

**Published:** 2025-03-31

**Authors:** Yong Chen, Yulang Xu, Xianrui Zhao, Yefang Wang, Wangwang Yu, Tao Zhang, Chenfu Fang

**Affiliations:** 1 School of Mechanical Engineering, Nanjing Vocational University of Industry Technology, No. 1 Yangshan North Road, Nanjing 210023, China; 2 School of Marine and Intelligent Manufacturing, Jiangsu Maritime Institute, No. 309 Gezhi Road, Nanjing 211100, China; 3 Provincial Key Lab of Advanced Welding Technology, School of Materials Science and Engineering, Jiangsu University of Science and Technology, No. 2 Mengxi Road, Zhenjiang 212003, China

**Keywords:** seven-wire, elelctrogas welding, toughened welding wire, microstructure, mechanical properties

## Abstract

Based on the flexible adjustment of the seven-wire, this study will assemble a new toughened seven-wire which is combined with a common single welding wire and the existing welding wire containing ductile alloy element (Ni element), and the microstructure properties, mechanical properties and toughening mechanism of the welding seams were studied. The results show that the microstructure of the four combinatorial seven-wire welding seams is mainly composed of coarse proeutectoid ferrite (PF) and fine acicular ferrite (AF). Among them, the core of inclusions that induce AF nucleation and growth are mainly composed of Al, Ti, Si, and Mn-based oxides, and the edge of inclusions is mainly composed of Mn and Cu sulfides (MnS, CuS). The addition of Ti compounds further promotes AF nucleation. This is also a reason why the impact toughness of the combinatorial seven-wire W2/W3 welding seams is higher than that of other combinatorial seven-wire welding seams, but the impact toughness of the rich Ni seven-wire can meet the standard requirements of the China Classification Society (CCS). Among the four combinatorial seven-wire welding seams, the proportions of large angle grain boundaries (grain orientation difference ≥ 15°) that improve the ability of materials to prevent brittle fracture are 65.9%, 68.8%, 66.0%, 61.7%, respectively, that is, the larger proportion of large angle grain boundaries in combinatorial seven-wire W2 welding seams (Ni content is 0.0897%) is one of the reasons for the higher impact toughness of the welding seams. With the increase of Ni content in the welding seam, the AF content first increased and then decreased, the yield strength and tensile strength increased, and the elongation and section shrinkage first increased and then decreased. When the combinatorial seven-wire W2/W3 was used, the welding seam plasticity was the best.

## 1. Introduction

Electrogas welding (EGW) is a high heat input efficient welding method mainly used in thick plate vertical seams, which is widely used in ships, pressure vessels, and other industries. Weld toughness is one of the important indicators to measure the quality of hot input welding joints [1,2,3,4]. At present, welding scholars have studied the influence of the welding process on weld toughness and the influence of alloy elements on weld toughness. Peng et al. use mechanical vibration to improve the weld toughness. The vibration accelerates the thermal movement of the metal atoms in the molten pool and increases the periodic external force (shock force) applied to the weldment during the orderly atomic welding process, thus reducing the welding residual stress and improving the welding quality. On the premise of ensuring the stability of the welding process, the higher the vibration frequency, the higher the percentage of the sample impact work. This is due to the decrease of the welding parts and the vibration of the particles and the improvement of the weld toughness gradually weakens with the decrease of the test temperature, and the influence of vibration on the weld toughness basically disappears when the test temperature drops to a certain value [5]. Guo et al. study the submerged arc welding of pipeline steel, and the longitudinal magnetic field parallel to the arc is used to realize electromagnetic stirring, and the impact toughness of the weld is studied for comparison. The results show that compared with the conventional pipeline steel submerged arc welding, the low-temperature toughness of the molten metal is increased by about 39%, which is because the electromagnetic stirring suppresses the formation of ferritons and the side plate at the grain boundary and increases the proportion of acicular ferrite (AF) in the crystal [6]. Yuan et al. apply the energy field including the electromagnetic vibration and ultrasonic vibration to weld the magnesium alloy. Both applied energy fields can refine the grain, thus increasing the weld toughness. The electromagnetic arc swing not only strengthens the flow of the molten pool but also plays the preheating effect on the weld, thus reducing the temperature gradient and increasing the component overcooling; the ultrasonic mixing directly introduces the ultrasonic energy into the magnesium alloy welding pool and refining the grains of the magnesium alloy weld, which breaks the tip at the moment of collapse. With the increase of the ultrasonic stirring amplitude and the distance between the ultrasonic probe and the arc, the refinement effect of the magnesium alloy weld is gradually enhanced, and the toughness of the weld is gradually increased [7,8]. Song et al. conducted an EGW test on the independently developed drug core welding wire, and the influence of different heat inputs on the molten metal tissue and impact toughness of the weld was studied. The results show that under the conditions of heat input welding, a large number of fine and diffuse inclusions are formed in the molten metal, and a large number of interlocked AF grains induced around the inclusion are the main reasons for the high and low-temperature impact toughness of the fused metal [9]. A et al. studied the influence of titanium and boron on the toughness of the weld. The results show that the content of these two elements in the weld has a parabolic influence on the toughness of the weld, that is, with the increase of both elements, the toughness of the weld joint first increases and then decreases. When the content of these two elements is within a certain range, a large number of fine AF are obtained in the weld, and the welded tissue and low-temperature toughness can be significantly improved. When the content is excessive, the AF in the weld is reduced, and the low-temperature toughness is reduced accordingly [10,11]. Jin et al. discuss the influence of light rare earth on the toughness of the weld. In the low-alloy high-strength steel welding rods, the light rare earth elements are transferred through the medicine skin. The results show that the appropriate amount of rare earth has the function of dehydrogenation, desulfurization, reducing the number of inclusions, and purifying the weld tissue. Rare earth can resist the transformation of ferrite on the side plate and promote the formation of needle ferrite, but when the amount of rare earth is too large, resulting in increased inclusion and loose tissue, which makes the toughness of the weld decrease [12].

To sum up, to increase the weld toughness, on the one hand, from the perspective of welding technology, by adding mechanical vibration devices, magnetic field devices, ultrasonic stirring devices, etc. to accelerate the flow of liquid metal in the molten pool, the microstructure of the weld seam is refined, and the toughness of the weld seam is increased; On the other hand, from the perspective of welding wire formulation, by adding toughening alloy elements to obtain a large amount of AF, the goal of increasing weld toughness can be achieved [13,14].

Seven-wire rotating arc (SRA) EGW is a new EGW process with seven-wire as the melting pole. In the welding process, the rotation of the arc and droplet can stir the molten pool, accelerate the flow velocity of liquid metal in the molten pool, and thus achieve the homogenization of liquid metal in the molten pool [15,16,17,18,19]. The seven-wire formed by rotating and twisting seven independent branch welding wires can be combined with different types of branch welding wires at will. Therefore, existing welding wires rich in toughening alloy elements can be used as branch welding wires and twisted with ordinary welding wires without changing the branch welding wire formula [20,21,22,23,24,25]. Through the chemical metallurgical reaction between the transition droplets and the base metal, the toughening alloy elements can be transferred to the weld seam, thereby increasing the toughness of the weld seam. This article will analyze the weld performance and study the toughening mechanism of a new type of toughened seven-wire welding wire, based on the flexible adjustment of the seven-wire welding wire. Some of the seven-wire welding wires will be replaced with existing ones containing toughening alloy elements (Ni elements) to form a new type of toughened seven-wire welding wire.

## 2. Materials and Methods

**Figure 1:**
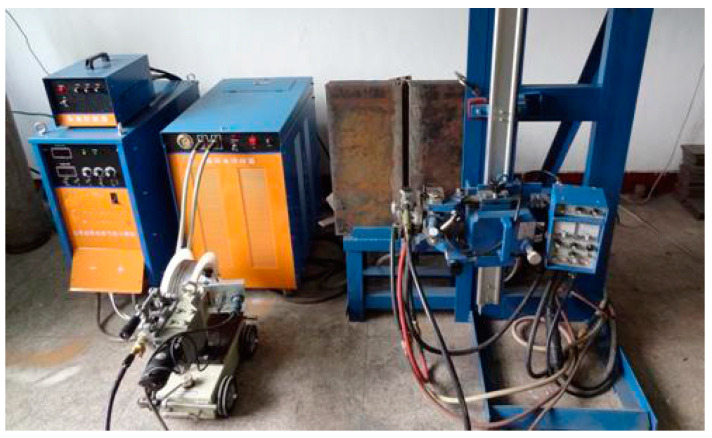
Diagram of the experimental setup.

Figure 1 shows the diagram of the experimental setup. The experimental system mainly consists of a welding power supply, coordination controller, wire feeder, welding gun, water cooling system, crawling system, water cooling slider, ceramic liner, and shielding gas.

The base metal employed in this study is AH36 marine-grade low-carbon high-strength steel. For the welding process, a mixed shielding gas consisting of 80% argon (Ar) and 20% carbon dioxide (CO_2_) was utilized, with the gas flow rate maintained at a constant 30 L/min throughout the welding operation. Φ2.4 mm seven wire is used for the EGW test. Φ2.4 mm seven-wire is made of 7 Φ 0.8 mm wire welding wire. The grades of the two welding wires are GB/T ER50-6 and GB/T ER55-Ni1 (produced by Jiangsu Lianjie Welding Technology Co., Ltd., Changzhou, China) respectively. The chemical composition and mechanical properties of the welding wire are shown in Table 1 and Table 2.

**Figure 2:**
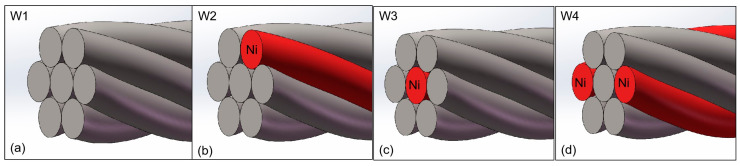
Schematic diagram of combinatorial seven-wire. (**a**) W1, (**b**) W2, (**c**) W3, (**d**) W4.

In order to study the best combination of toughness seven-wire, the ordinary welding wire is the main welding wire, and several Ni solid welding wires. The schematic diagram of the seven filaments is shown in Figure 2. In Figure 2a, seven ordinary solid welding wires are twisted as W1. In Figure 2b, six ordinary solid welding wires and a rich Ni solid welding wire are recorded as W2. In Figure 2c, six ordinary solid welding wires and a rich Ni solid welding wire are twisted as W3. In Figure 2d, five ordinary solid welding wires and two rich Ni solid welding wires as symmetrical peripheral welding wires are recorded as W4.

Table 3 shows the chemical composition of the weld seams of four combinatorial seven-wire (W1, W2, W3, and W4) analyzed using a PDA-5000 direct reading spectrometer. According to Table 3, all four combinatorial seven-wire welds contain the same alloy elements. As the number of rich Ni filler wires in the combination increases from 1 to 2, the content of some alloy elements in the combinatorial seven-wire welds decreases, such as C and Mn elements. This is because the corresponding alloy element content in the rich Ni filler wire is lower than that in the ordinary filler wire. Correspondingly, the Ni element content in the transition to the weld increases with the increase of the number of Ni-rich filler wires, from 0.0288% to 0.1547%. The chemical composition of the weld seam of combinatorial seven-wire W2 and W3 is similar. This is because although the combination methods of the two combinatorial seven-wire are different, the use of separate welding wires is the same. The difference in the content of some alloy elements in the weld seam is caused by the differences in the chemical composition of their respective separate welding wires.

**Figure 3:**
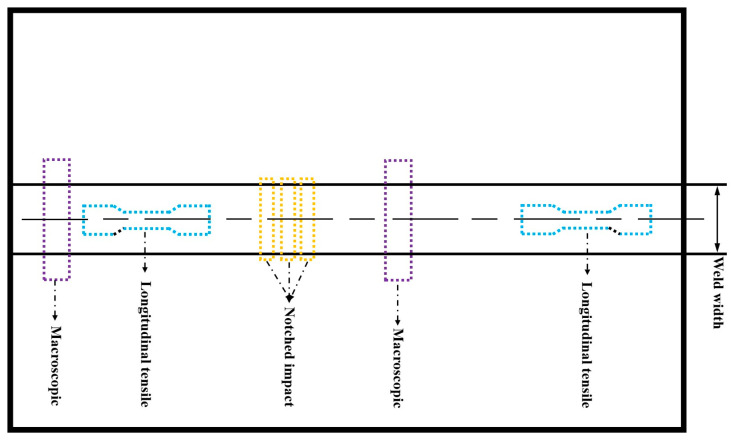
Schematic diagram of mechanical properties specimens of butt joint in SRA EGW.

The welding joint specimens are subjected to water grinding first, followed by polishing with 3.5 µm diamond polishing paste. After polishing, the specimens are etched using a 5% nitric acid alcohol solution, followed by cleaning with alcohol, and then dried with hot air. The prepared metallographic samples are observed under an optical microscope (Leica MM6) for microstructural analysis. Further observation of the weld joint’s microstructure is conducted using a tungsten filament scanning electron microscope (SEM) model JSM-8680, produced by JEOL. Observation of microstructure of welded joints using JEM-2001F field emission lens electron microscope (TEM) produced by Nippon Electric Corporation. The hardness test is conducted using the Vickers hardness method, with a test load of 10 kg and a holding time of 15 s. According to the Rules for Materials and Welding by the China Classification Society (CCS), the mechanical properties of welded joints are primarily analyzed through longitudinal tensile, impact tests. The specimens for these tests are cut from butt-welded test pieces. The schematic diagram of mechanical properties specimens of butt joints in EGW is shown in Figure 3.

## 3. Results and Discussion

### 3.1. Microstructure Analysis

Compared to single-wire welding wire, the seven-wire design obtains a large amount of AF structure by adding toughening alloy elements. AF is the most desirable ideal structure for low-alloy high-strength steel welds, with good strength, toughness, and crack propagation resistance. If a large amount of AF can be obtained, it will greatly improve the performance of the weld. The reason is that there is a high angle grain boundary between AF Flat noodles, and AF dislocation density is high. The microcrack cleavage across AF will consume a lot of energy, which will help to improve the toughness of welded joints while improving the strength. In addition, in SRA EGW, the rotation of the arc and droplets can stir the molten pool, accelerate the flow velocity of the liquid metal in the molten pool, and thus achieve the homogenization of the liquid metal in the molten pool.

**Figure 4:**
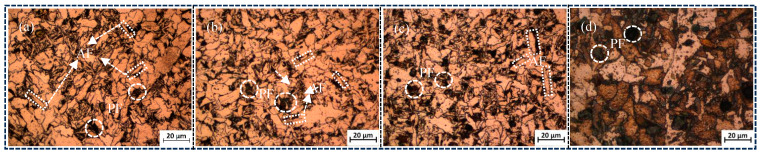
Microstructures of weld seams with four combinatorial seven-wire. (**a**) W1, (**b**) W2, (**c**) W3, (**d**) W4.

**Figure 5:**
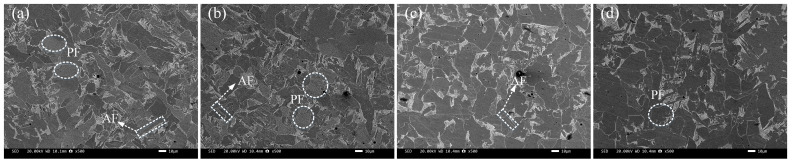
SEM microscopic morphology of weld seams with four combinatorial seven-wire. (**a**) W1, (**b**) W2, (**c**) W3, (**d**) W4.

The microstructure of different seven-wire combination welds is shown in Figure 4a–d, as well as Figure 5a–d. The microstructure of the combination of seven-wire W1, W2, and W3 welds is mainly composed of coarse pre-eutectoid ferrite (PF) and AF. PF is characterized by elongated and polygonal shapes along the grain boundaries, while AF is mainly formed within the austenite grains and grows radially with inclusions as nucleation centers. The aspect ratio is generally in the range of 3:1 to 10:1. When the combination of seven-wire is twisted using five ordinary solid welding wires and two Ni-rich solid welding wires as symmetrical peripheral parting wires, as shown in Figure 4d, the microstructure of the W4 weld seam of the combination of seven-wire is mainly composed of PF. At this time, the PF becomes coarser and has a larger area.

**Figure 6:**
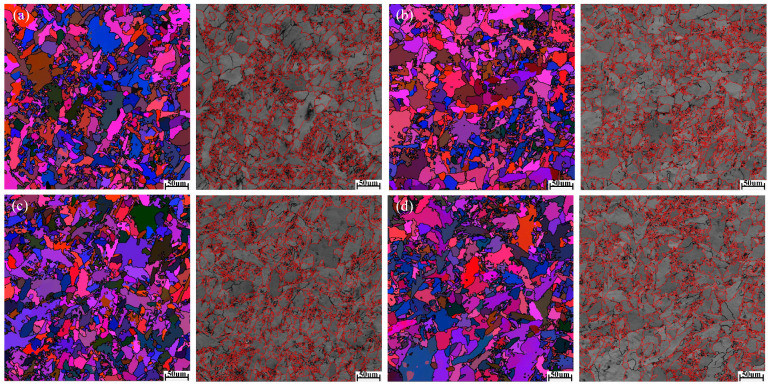
EBSD images of weld seams with four combinatorial seven-wire. (**a**) W1, (**b**) W2, (**c**) W3, (**d**) W4.

Figure 6a–d shows the EBSD Euler plots and grain boundary type distribution maps of the welded joints with four combinatorial seven-wire W1, W2, W3, and W4, respectively. Each color in the figure represents a grain orientation, and as shown in Figure 6, the microstructure of the four combinatorial seven-wire welds includes coarse PF and fine AF.

**Figure 7:**
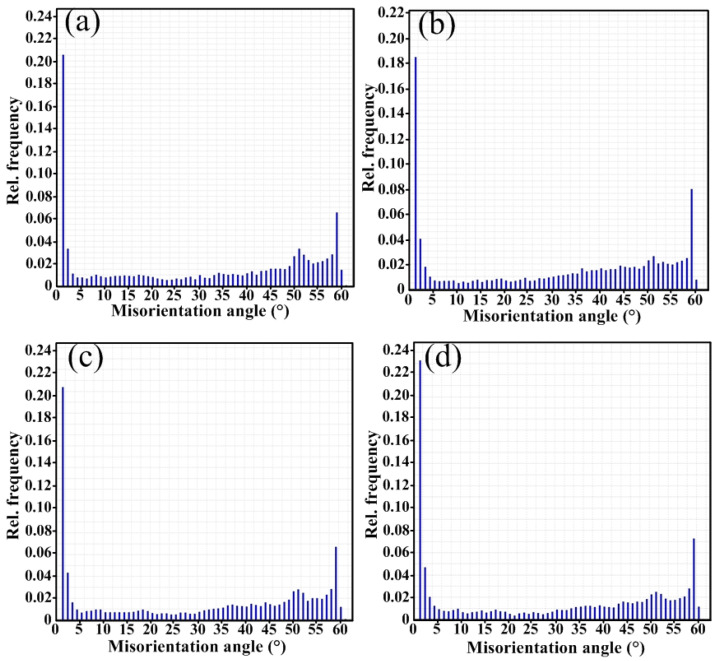
Misorientation angle of weld seams with four combinatorial seven-wire. (**a**) W1, (**b**) W2, (**c**) W3, (**d**) W4.

In Figure 6a–d, red represents large angle grain boundaries greater than 15°, and black represents small angle grain boundaries between 2° and 15°. The distribution of grain orientation differences in the four combinatorial seven-wire welds was statistically analyzed, and the results are shown in Figure 7. Due to the hindering effect of large angle grain boundaries (grain orientation difference ≥ 15°) on crack propagation, when cracks encounter large angle grain boundaries during the propagation process, the direction of the crack propagation path changes, making the propagation path tortuous and consuming more energy, which can improve the material’s ability to prevent brittle fracture. Therefore, increasing the proportion of large-angle grain boundaries can improve impact toughness. The proportion of large angle grain boundaries in the weld obtained by using the combinatorial seven-wire W1, W2, W3, and W4 is 65.9%, 68.8%, 66.0%, and 61.7%, respectively. It can be seen that the proportion of large-angle grain boundaries in the weld obtained by using the combinatorial seven-wire W2 is the highest, while the proportion of large-angle grain boundaries in the weld obtained by using the combinatorial seven-wire W4 is the lowest. That is, the larger proportion of large angle grain boundaries in the weld obtained by using the combinatorial seven-wire W2 is one of the reasons why its impact toughness is higher than that of the weld obtained by using the combination of ordinary welding wire W1.

**Figure 8:**
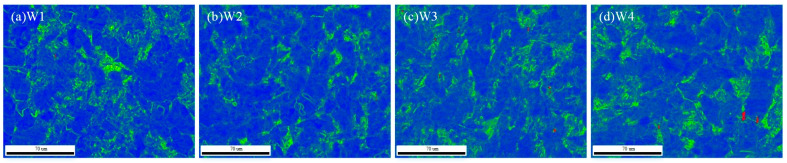
KAM map of weld seam area with different combinatorial seven-wire.

Figure 8a–d shows the KAM (Kernel Average Misorientation) drawing. The green area indicates the dislocation density, and it can be seen that when the W2 welding wire is selected, the percentage of the green area is small, indicating that the dislocation density is low, and the weld presents better toughness characteristics.

**Figure 9:**
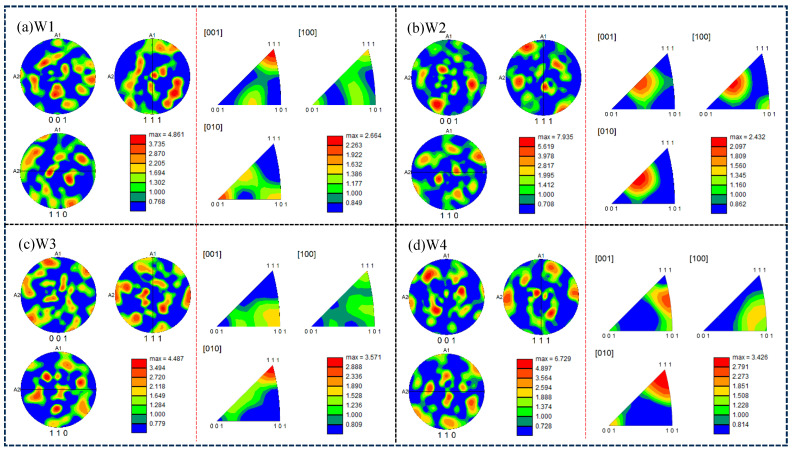
Polar and inverse polar diagrams of weld seam area with different combinatorial seven-wire.

**Figure 10:**
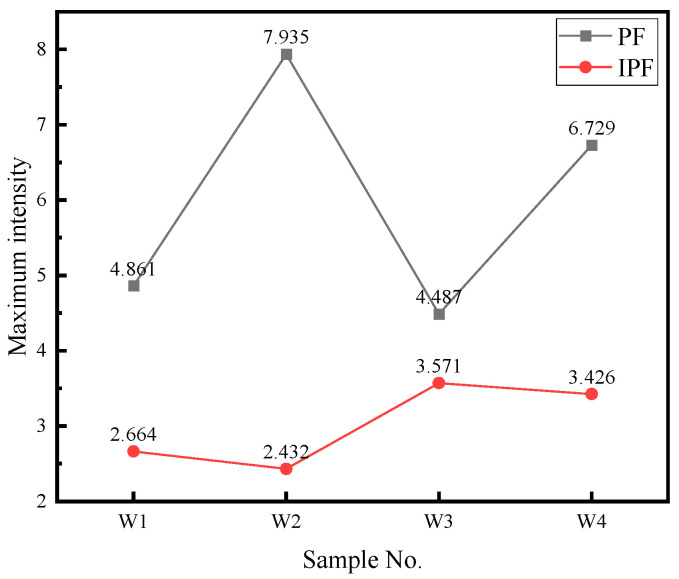
Maximum pole density of welds under different combinatorial seven-wire.

Figure 9a–d are the pole and reverse pole maps of the weld area under different combinatorial seven-wire, and the maximum electrode density of the corresponding four combinatorial seven-wire is shown in Figure 10. The maximum pole density is 7.935 in W2 and the reverse is 2.432.

**Figure 11:**
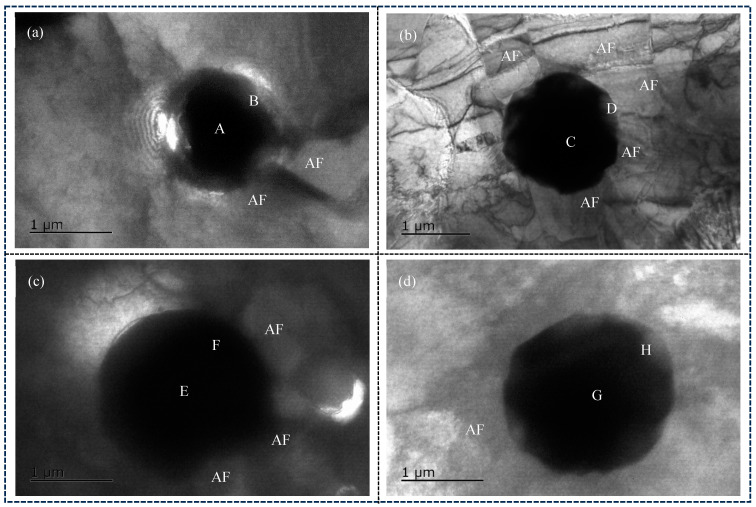
TEM image analyses in welding seam with different combinatorial seven-wire. (**a**) W1, (**b**) W2, (**c**) W3, (**d**) W4.

Figure 11a–d shows the TEM images of inclusion in the combinatorial seven-wire W2 weld, and the results of atomic content (at. %) in EDS are shown in Table 4.

As shown in Figure 11a, the inclusions in the weld obtained by using the combinatorial seven-wire W1 are spherical, and two AF are induced around the inclusions. EDS analysis was performed on the center point A and edge point B of the inclusions, and the results showed that the main element of the inclusions is Mn-Si-Al-O. These inclusions may be composed of Mn-based oxides, Si-based oxides, and Al-based oxides. Further observation of the figure revealed the presence of small amounts of Ti, Cu, and S elements in the inclusions, indicating the presence of titanium oxides (TiO, Ti_2_O_3_, TiO_2_, and TiN), copper sulfide, and manganese sulfide in the inclusions. Due to the small size of inclusions, the electron beam of transmission electron microscopy may hit noninclusion parts and be affected by them. Therefore, the Fe element in the inclusion EDS analysis composition table is not the chemical composition element of inclusions, and the same applies to the following text. As shown in Figure 11b, the inclusion is also spherical, and five intersecting and cross-shaped AF are induced around the inclusion. EDS analysis was performed on the center point A and the right edge point B of the inclusion, and the results showed that the main element of the inclusion is Mn-Si-Al-O, and the inclusion may be composed of oxides of these elements. According to Figure 11c, three AF are induced around the spherical inclusion. EDS composition analysis was performed on the center and edges of the inclusion, and the results showed that the main element of the inclusion was Mn-Ti-Si-Cu-S-O, which means that the inclusion is mainly composed of oxides of various elements and sulfides of Mn and Cu. According to Figure 11d, an AF is induced around the spherical inclusion. EDS composition analysis of the center and edges of the inclusion shows that the main element of the inclusion is Mn-Cu-Ti-Si-O, which means that the inclusion is mainly composed of oxides of various elements.

**Figure 12:**
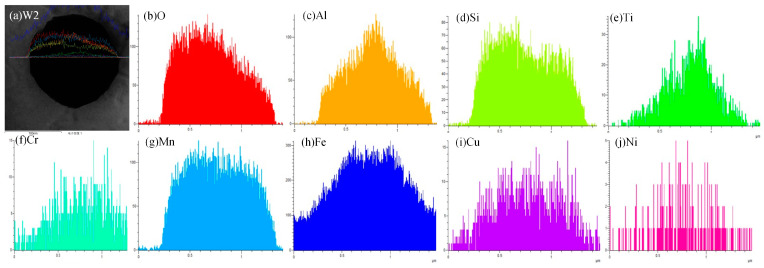
Elements distribution across the inclusion in weld seam with W2.

**Figure 13:**
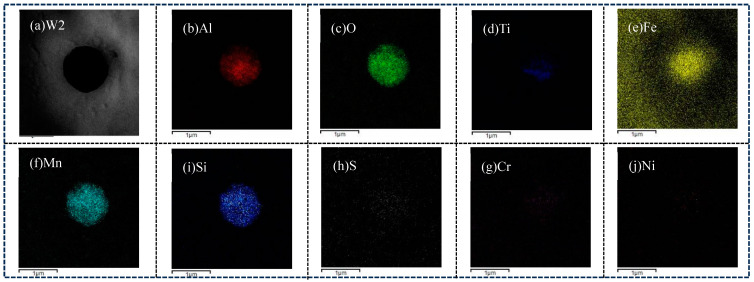
EDS-mapping result of inclusion in weld seam with W2.

For W2, in order to further analyze the distribution of various elements on the inclusion, EDS line scanning and surface scanning methods were used to analyze the inclusion, and the results are shown in Figure 12 and Figure 13, respectively. As shown in Figure 12 and Figure 13, the main element at the center of the inclusion is Mn-Si-Al-Ti-O. Therefore, in addition to Mn-based oxides, Si-based oxides, and Al-based oxides, there are also Ti-based compounds (Ti-based oxides and TiN) at the center of the inclusion. Ti is more conducive to promoting the nucleation of AF compared to Mn, and Ti-based compounds can even precipitate on already-formed oxide inclusions and become nucleation particles of AF. Compared with inclusions composed only of oxides, it has a better effect on promoting the nucleation of AF; At the edge of the inclusion, the main element is Mn-Si-Cu-S, with very few Al and O elements. Therefore, at the edge of the inclusion, the main elements are Mn-based and Cu-based sulfides, as well as Si-based oxides, namely MnS, CuS, SiO_2_, and trace amounts of Al-based oxides.

**Figure 14:**
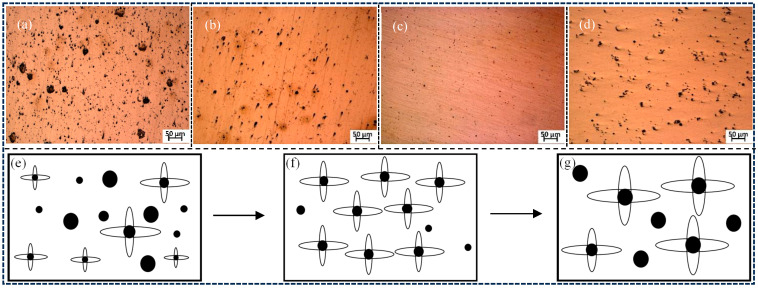
Inclusions distribution and mechanism of nucleation variation in weld seam with four combinatorial seven-wire. (**a**) W1, (**b**) W2, (**c**) W3, (**d**) W4, (**e**) nucleation variation 1, (**f**) nucleation variation 2, (**g**) nucleation variation 3.

Figure 14a–d shows the distribution of inclusions in the weld seams of combinatorial seven-wire W1, W2, W3, and W4, respectively. It can be seen that the inclusions in the weld seams of the four combinatorial seven-wire are mainly spherical. The combinatorial seven-wire W1 weld has the most inclusions. On the one hand, there are many small diameter inclusions as nucleation sites, which are beneficial for the impact toughness of the weld. However, the presence of larger diameter inclusions in the weld reduces its toughness, resulting in lower impact toughness of the combinatorial seven-wire W1 weld. When using the combinatorial seven-wire W2, the number of inclusions in the weld seam decreases, the overall size of inclusions decreases, and there are no large diameter inclusions. Therefore, the presence of more small diameter inclusions that are beneficial for impact toughness increases the impact toughness of the weld seam. When using the combinatorial seven-wire W3, the number of inclusions in the weld seam is further reduced, but the overall size of inclusions is greatly reduced and the distribution is more uniform, thereby ensuring the improvement of the impact toughness of the weld seam. When using the combinatorial seven-wire W4, the number of inclusions decreases continuously while the overall size increases, resulting in the formation of more large-diameter inclusions, thereby reducing the impact toughness of the weld.

It can be seen that the impact toughness in the weld is not only related to the number of inclusions in the weld but also to the size of the inclusions. The significant strain energy caused by inclusions in their vicinity is one of the important factors in AF nucleation, but as an inert medium surface, inclusions play a decisive role in inducing AF nucleation. It should be noted that although the number of inclusions in the weld seam is reduced when using the combinatorial seven-wire W2 and W3, there are more small-sized inclusions that can serve as nucleation points for AF. The good lattice matching between AF and inclusions reduces the nucleation activation energy, thereby ensuring an increase in AF content in the combinatorial seven-wire W2 and W3 weld seam and improving the impact toughness of the weld seam. The mechanism of its changes is shown in Figure 14e–g. In summary, inclusions are conducive to the non-uniform nucleation of needle-like ferrite, which then grows in multiple directions from this nucleation point. Due to its chain structure, it can effectively prevent crack propagation, resulting in a significant improvement in the toughness of the weld.

Furthermore, due to the same welding conditions when using four different combinatorial seven-wire welding, the different proportions of AF in each combinatorial seven-wire weld are mainly related to the chemical composition of the respective welding wires, and the addition of alloying elements plays an important role in controlling their microstructure. According to Table 3, all four combinatorial seven-wire welds contain alloying elements that promote the formation of AF, such as Ti B, Mo, Cr, Al, Ni, and Mn. The main purpose of adding the above alloying elements is to suppress the formation of PF in the weld seam. With the increase of the above alloying elements, their hindering effect on the precipitation of PF is further enhanced, thereby continuously delaying the transformation of austenite to PF in the weld seam, and thus the weld seam can obtain more intermediate temperature transformation structures (AF or bainite). Further observation revealed that, except for the significant change in Ni content, the content of other alloy elements in the four types of combined seven-wire welds did not change much. Therefore, it can be inferred that different microstructures were obtained in the four types of combinatorial seven-wire welds, especially the different AF ratios in the microstructures, which are mainly related to the different Ni content in each weld. Ni is an austenitizing element that can increase the stability of austenite in welds, reduce the transition temperature from γ-Fe to α-Fe in welds, decrease the content of PF, and increase the content of AF. At the same time, the presence of Ni in welds can promote cross slip, thereby increasing weld toughness. However, when Ni is too high, the microstructure grains in welds become coarse, reducing weld toughness.

### 3.2. Mechanical Properties Analysis

**Figure 15:**
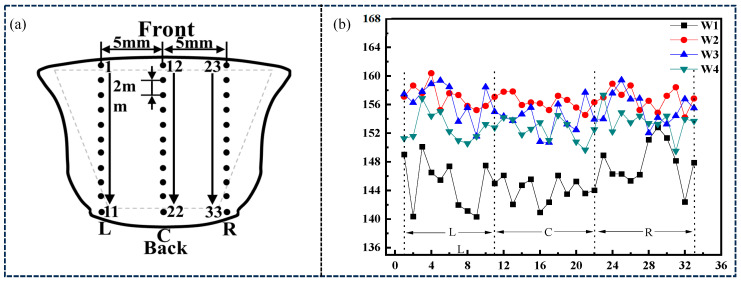
Hardness test of welding seam with four combinatorial seven-wire. (**a**) distribution of Vickers hardness test points, (**b**) Vickers hardness test results.

In order to analyze the distribution of overall hardness values of the welds, the centers of the front and back welds were taken as the centerline of the hardness test, with 5 mm on each side. Hardness spot tests were conducted along the plate thickness direction from the front weld to the back weld, with 11 test points in each column. Therefore, there are a total of 33 hardness test points in each weld. The distribution of Vickers hardness test points is shown in Figure 15a, and the test results are shown in Figure 15b.

As shown in Figure 15b, the average hardness of the L column in the combinatorial seven-wire W1 weld is 144.97 HV10, the average hardness of the C column is 144.02 HV10, the average hardness of the R column is 147.89 HV10, and the overall hardness of the weld is 145.63 HV10; The average hardness of the L column in the combinatorial seven-wire W2 is 157.07 HV10, the average hardness of the C column is 156.32 HV10, the average hardness of the R column is 156.84 HV10, and the overall hardness is 156.74 HV10; The average hardness of the L column in the combinatorial seven-wire W3 weld is 156.58 HV10, the average hardness of the C column is 153.92 HV10, the average hardness of the R column is 155.52 HV10, and the overall hardness of the weld is 154.80 HV10; The average hardness of the L column in the combinatorial seven-wire W4 weld is 152.77 HV10, the average hardness of the C column is 152.52 HV10, the average hardness of the R column is 153.68 HV10, and the overall hardness is 152.99 HV10. From the analysis above, it can be seen that the Vickers hardness values of the three columns near the left side wall, the center C column of the weld, and the L and R columns near the two side walls in the four combinatorial seven-wire welds do not change much. This may be related to the unique rotating arc of the seven-wire weld during the welding process, that is, the rotation of the arc causes the heat in the molten pool to diffuse more evenly around the molten pool, resulting in a more uniform microstructure of the weld. Further observation shows that the average hardness value of the weld near the two side walls is slightly higher than that of the center C column of the weld, but the difference is not significant, that is, the average hardness value of the weld at the center C column of each weld is the lowest, and the hardness value of the weld near the two side walls is slightly higher. This is because although the rotation of the arc improves the heat of the liquid Compared to the diffusion on both sides of the wall, The heat on the center C column of the weld is still greater than the heat near the two side walls, resulting in a coarser microstructure on the center C column of the weld (mainly PF), which slightly reduces the hardness value. It should be noted that as the Ni content in the weld increases, from 0.0288% to 0.1547%, the average Vickers hardness of the weld first increases and then decreases. That is, when using combinatorial seven-wire W3, the average Vickers hardness of the weld is the highest, and when using combinatorial seven-wire W1, the average Vickers hardness of the weld is the lowest.

**Figure 16:**
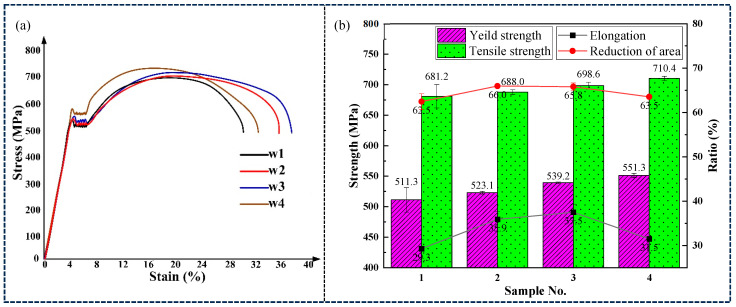
Tensile test of weld seams with four combinatorial seven-wire. (**a**) stress-strain curves of the weld tensile specimens, (**b**) tensile test results.

Longitudinal tensile tests were conducted on four combinatorial seven-wire welds. The stress-strain curves of the weld tensile specimens are shown in Figure 16a, and the corresponding tensile test results are shown in Figure 16b. With the increase of Ni content in the four combinatorial seven-wire welds, both the yield strength and tensile strength of the welds increase. The yield strength increases from 511.3 MPa in the combinatorial seven-wire W1 welds to 551.3 MPa in the combinatorial seven-wire W4 welds, with an increase of 7.8%. The tensile strength increased from 681.2 MPa for the combinatorial seven-wire W1 weld to 710.4 MPa for the combinatorial seven-wire W4 weld, with an increase of 4.3%. As the Ni content in the four combinatorial seven-wire welds increases, both the weld elongation and the weld section shrinkage first increase and then decrease. When the Ni content increases from 0.0288% to 0.0917%, the weld elongation increases from 29.3% to 37.5%, an increase of 27.9%, and the weld section shrinkage increases from 62.5% to 65.8%, an increase of 5.4%. As the Ni content increases to 0.1547%, the weld elongation decreases to 31.5% and the weld section shrinkage decreases to 63.5%.

**Figure 17:**
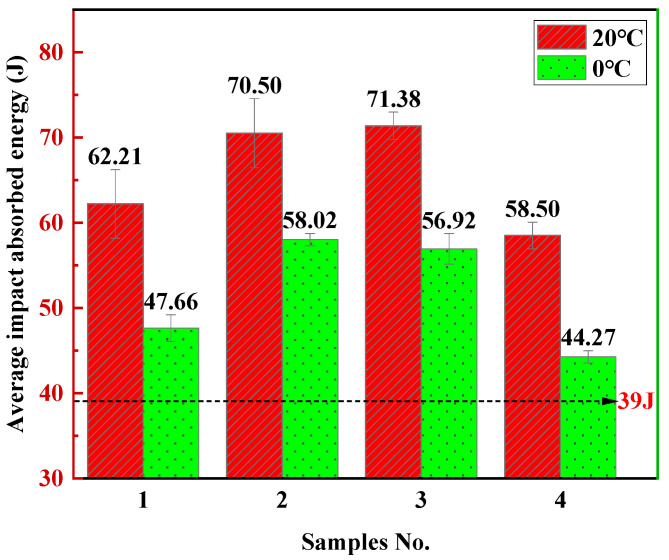
Impact test results of weld seams with four combinatorial seven-wire.

Six impact toughness specimens were taken from four sets of welded joints, and each group of three specimens was subjected to weld impact toughness tests at 20 °C and 0 °C. The results of four combinatorial seven-wire weld impact tests are shown in Figure 17. At 20 °C, the highest average impact energy of the weld seam is 70.50–71.38 J (W2, W3), which is 13.3% higher than the average impact energy of the weld seam of the seven wire W1 composed of ordinary welding wires (62.21 J). At 0 °C, the highest average impact energy of the weld seam decreased to 56.92–58.02 J (W3, W2), which was 19.4% higher than the average impact energy of the weld seam of the seven-wire W1 composed of ordinary parting wires (47.66 J). Through observation, it was found that as the experimental temperature decreases, the average impact energy of all four combinatorial seven-wire welds decreases accordingly. This is because as the temperature decreases, the welds gradually exhibit brittle characteristics, but the variation pattern of the average impact energy of the four combinatorial seven-wire welds is similar. It should be noted that the average impact energy of the welds using the combinatorial seven-wire W1, W2, W3, and W4 is higher than the 39 J required by the China Classification Society (CCS) standard at both temperatures. Among them, the average impact energy of the welds using the combinatorial seven-wire W2/W3 is higher than the average impact energy of the welds using the other combinatorial seven-wire at the same temperature. Further observation revealed that the average impact energy of the four combinatorial seven-wire welds under 20 °C and 0 °C conditions showed a pattern of W2/W3 > W1 > W4.

Based on the alloy element content of the four combinatorial seven-wire in Table 3 and the microstructure analysis in Figure 4 and Figure 5, it can be seen that with the increase of Ni content in the weld, the AF content in the weld first increases, and then decreases. That is, when the Ni content in the weld increases from 0.0288% to 0.0917%, the AF content in the weld microstructure is the highest, and the corresponding weld impact toughness value reaches the maximum. With the further increase of Ni content in the weld, that is, when the Ni content increases to 0.1547%, the PF content not only increases but also becomes coarser, while the AF content sharply decreases. The AF ratio in the four combinatorial seven-wire welds reflects the impact toughness performance of each weld, that is, more AF ratios in the weld are also one of the reasons for the higher impact toughness of the combinatorial seven-wire W2/W3 weld. It should be noted that the joint action of Ni and Mn elements in the weld seam will constrain each other, thereby affecting the microstructure of the weld seam. When the proportion of the two elements in the weld seam is not appropriate, the formation of needle-like ferrite in the weld seam will be suppressed, thereby reducing the toughness of the weld seam. Under the experimental conditions in this article, when the Mn content in the weld seam is within the range of 1.107~1.239%, the optimal Ni content is 0.897~0.917%.

**Figure 18:**
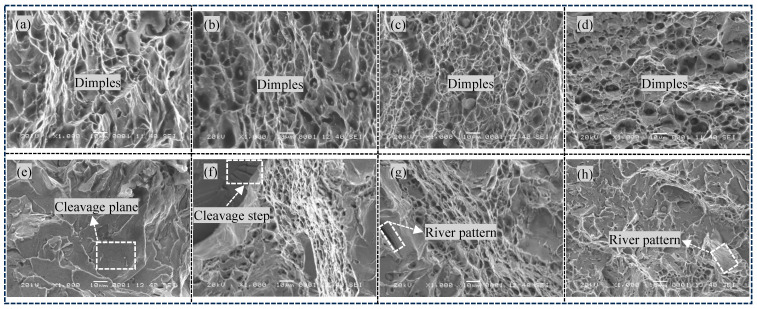
Impact fracture SEM of weld seams with four combinatorial seven-wire. (**a**) W1 at 20 °C, (**b**) W2 at 20 °C, (**c**) W3 at 20 °C, (**d**) W4 at 20 °C, (**e**) W1 at 0 °C, (**f**) W2 at 0 °C, (**g**) W3 at 0 °C, (**h**) W41 at 0 °C.

Figure 18 shows SEM images of the impact fracture surfaces of four combinatorial seven-wire welds at different temperatures. Figure 18a–d shows typical SEM images of the impact fracture surfaces of the combinatorial seven-wire W1 to W4 welds at 20 °C. It can be seen that the impact fracture surfaces of the combinatorial seven-wire W1, W2, W3, and W4 welds have obvious dimples. Comparison shows that the distribution of dimples on the fracture surfaces of the W2/W3 specimens is more uniform and deeper, while the dimples on the fracture surfaces of the W4 specimen show obvious small and shallow characteristics.

As the impact test temperature decreased to 0 °C, the impact fracture surfaces of these four combinatorial seven-wire welds clearly changed from ductile fracture to quasi-cleavage fracture, as shown in Figure 18e–h. The fracture surface presents a clear river-like pattern, and there are tearing edges around the quasi-cleavage plane, as well as a large number of ductile dimples. Due to the fact that the connection between quasi-cleavage planes is mainly achieved through shear or tearing with large plastic deformation, the formation process is similar to that of ductile dimples, forming local ductile dimples or tearing edges around them. Because quasi-cleavage fracture is caused by the propagation of many individual nucleated microcracks, which are connected to each other in a tearing manner and ultimately form a fracture, the quasi-cleavage facets are in a micro concave shape.

## 4. Conclusions

(1) The microstructure of four combinatorial seven-wire welded joints is mainly composed of coarse PF and fine AF. Among them, the centers of inclusions that induce the nucleation and growth of AF are mainly composed of Al-based oxides, Ti-based oxides, Si-based oxides, and Mn-based oxides. At the edges of inclusions, they are mainly composed of Mn and Cu sulfides (MnS, CuS). The addition of Ti-based compounds further promotes the nucleation of AF.
(2) Among the four combinatorial seven-wire welds, the proportion of large angle grain boundaries (grain orientation difference ≥ 15°) that improve the material’s ability to prevent brittle fracture is 65.9%, 68.8%, 66.0%, and 61.7%, respectively. That is, the larger proportion of large angle grain boundaries in the combinatorial seven-wire W2 welds (Ni content of 0.0897%) is one of the reasons for its higher impact toughness.
(3) The impact toughness of combinatorial seven-wire W2 and W3 can meet the standard requirements of CCS (39 J). With the increase of Ni content in the weld, the AF content in the weld increases first and then decreases, the yield strength and tensile strength of the weld increase, while the weld elongation and section shrinkage increase first. In the combinatorial seven-wire W2/W3, the weld plasticity is the best.

## Figures and Tables

**Table 1:** Chemical composition of wires (wt.%).

Wires	C	Mn	Si	Cr	Ni	Mo	P	S	Cu	V	Rest
GB/T ER50-6	0.06–0.15	1.40–1.85	0.80–1.15	≤0.15	≤0.15	≤0.15	≤0.025	≤0.025	≤0.50	≤0.03	-
GB/T ER55-Ni1	≤0.12	≤1.25	0.40–0.80	≤0.15	0.80–1.10	≤0.35	≤0.025	≤0.025	≤0.35	≤0.05	≤0.50

**Table 2:** Mechanical properties of wires.

Wires	Tensile Strength (MPa)	Yield Strength (MPa)	Elongation (%)	Impact Absorbed Energy (J)
GB/T ER50-6	≥500	≥420	≥22	≥27 (−30 °C)
GB/T ER55-Ni1	≥550	≥470	≥24	≥27 (−45 °C)

**Table 3:** Mechanical properties of welding seams with four combinatorial seven-wire (wt.%).

Element	W1	W2	W3	W4
C	0.1120	0.1070	0.1080	0.1043
Si	0.469	0.420	0.420	0.462
Mn	1.239	1.201	1.198	1.107
P	0.0150	0.0152	0.0152	0.0151
S	0.0112	0.0118	0.0115	0.0113
Cu	0.0174	0.0194	0.0174	0.0173
Al	0.0156	0.0190	0.0153	0.0244
Ni	0.0288	0.0897	0.0917	0.1547
Cr	0.0507	0.0517	0.0500	0.0498
Ti	0.0055	0.0085	0.0074	0.0115
V	0.0023	0.0021	0.0017	0.0017
B	0.0016	0.0024	0.0025	0.0021
Mo	0.0015	0.0023	0.0017	0.0016

**Table 4:** Element content of characteristic phase.

Element (at. %)	O	Al	Si	S	Ti	Cr	Mn	Fe	Cu	Ca	Ni
A	44.97	11.02	16.77	0.45	1.05	0.23	23.24	1.73	0.55	—	—
B	51.92	10.14	14.85	0.43	0.86	0.17	18.85	2.28	0.42	0.07	—
C	44.57	15.29	6.87	0.12	0.64	0.37	7.79	22.44	0.73	—	—
D	38.00	10.89	9.09	0.19	0.50	0.46	10.74	29.24	0.88	—	
E	5.28	1.67	3.92	2.60	3.13	0.85	33.43	47.59	1.67	—	0.06
F	3.12	1.14	3.32	1.02	1.41	0.91	24.64	62.84	1.58	—	0.01
G	0.38	1.90	0.08	1.67	—	0.93	15.85	77.43	1.75	—	—
H	2.22	0.59	2.13	0.10	1.67	0.95	16.50	74.11	1.75	—	—

## Data Availability

The original contributions presented in the study are included in the article, further inquiries can be directed to the corresponding authors.
